# The burden, correlates and outcomes of left ventricular hypertrophy among young Africans with first ever stroke in Tanzania

**DOI:** 10.1186/s12872-021-02297-8

**Published:** 2021-10-09

**Authors:** Sarah Shali Matuja, Patricia Munseri, Candida Moshiro, Khuzeima Khanbhai, Karim Mahawish

**Affiliations:** 1 Department of Internal Medicine, Catholic University of Health and Allied Sciences, P.O Box 1464, Mwanza, Tanzania; 2 Department of Internal Medicine, Muhimbili University of Health and Allied Sciences, Dar es Salaam, Tanzania; 3 Department of Epidemiology and Biostatistics, Muhimbili University of Health and Allied Sciences, Dar es Salaam, Tanzania; 4 Department of Cardiology, Jakaya Kikwete Cardiac Institute, Dar es Salaam, Tanzania; 5 Department of Internal Medicine, Midcentral District Health Board, Palmerston North, New Zealand

**Keywords:** Left ventricular hypertrophy, Young adults, Hypertension, Echocardiography, Electrocardiography

## Abstract

**Background:**

Left ventricular hypertrophy is a pathophysiological response often due to chronic uncontrolled hypertension. Our primary aim was to investigate the magnitude, correlates and outcomes of left ventricular hypertrophy as a surrogate maker for chronic uncontrolled hypertension in young adults ≤ 45 years with stroke. Our secondary aim was to determine the accuracy of electrocardiography using Sokolow-Lyon and Cornell criteria in detecting left ventricular hypertrophy compared to echocardiography.

**Methods:**

This cohort study recruited young strokes who had undergone brain imaging, electrocardiography and transthoracic echocardiography at baseline. The modified Poisson regression model examined baseline correlates for left ventricular hypertrophy. The National Institute of Health Stroke Scale assessed stroke severity and the modified Rankin Scale assessed outcomes to 30-days. Performance of electrical voltage criterions was estimated using receiver operator characteristics.

**Results:**

We enrolled 101 stroke participants. Brain imaging revealed ischemic strokes in 60 (59.4%) and those with intracerebral hemorrhage, 33 (86.8%) were localized to the basal ganglia. Left ventricular hypertrophy was present in 76 (75.3%:95%CI 65.7%–83.3%), and 30 (39.5%) and 28 (36.8%) had moderate or severe hypertrophy respectively. Young adults with premorbid or a new diagnosis of hypertension were more likely to have left ventricular hypertrophy, 47 (61.8%), and 26 (34.2%). On multivariable analysis, left ventricular hypertrophy was independently associated with not being on anti-hypertensive medications among hypertensives participants {adjusted risk ratio 1.4 (95%CI:1.04–1.94). The mean National Institute of Health Stroke score was 18 and 30-day mortality was 42 (43.3%). The sensitivity and specificity for Sokolow-Lyon in detecting left ventricular hypertrophy was 27% and 78%, and for Cornell was 32% and 52% respectively.

**Conclusions:**

We identified a high proportion of left ventricular hypertrophy in young adults with stroke associated with chronic undertreated hypertension. While the study methodology does not allow us to determine causation, this association and knowledge of pathophysiological processes supports the notion that chronic hypertension is a major risk factor for young strokes associated with high mortality. Our findings did not support the use of the electrical voltage criteria for detecting left ventricular hypertrophy. We recommend low cost interventions like blood pressure screening and treatment to reduce this burden.

## Background

Stroke is the leading cause of death and disability in low and middle income countries (LMIC), and accounts for 80% of the global burden [1]. Stroke incidence rises with age and though less common in the young, in this age group it is associated with particularly devastating effects on family and losses to the national economy. Cardiovascular diseases, particularly hypertension, is the leading risk factor for stroke in both high and LMIC, responsible for 20–50% of all strokes [2–4]. In sub Saharan Africa (SSA), 50% of stroke in the young is attributed to uncontrolled hypertension [5] and 45% of all stroke cases could be prevented by adequate blood pressure control [6, 7].

Uncontrolled chronic hypertension leads to target end organ damage, one manifestation of which is left ventricular hypertrophy (LVH). This occurs as a pathophysiological adaption to chronic increased afterload and serves as an independent predictor for coronary events, heart failure, ventricular arrhythmias, stroke and peripheral arterial disease [8, 9]. In SSA, there is a high burden of LVH (up to 50%) particularly among untreated hypertensive patients [10]. A study in West Africa revealed that more than half of the stroke patients were found to have LVH by electrocardiogram: independent predictors for LVH included younger age (< 45 years), female gender and uncontrolled hypertension [11]. Transthoracic echocardiography (TTE) is a noninvasive modality of choice to assess cardiac structure and function (including the detection of LVH) [12]. It is considered gold standard and superior to the 12-lead electrocardiogram (ECG) for diagnosing LVH [13].

We previously discovered high rates of hypertension (premorbid and new) in young stroke patients at our institution [5]. However, the role of hypertension in young strokes is unknown since; firstly, elevations in blood pressure is common following an acute stroke. Secondly, a premorbid history of hypertension may not be causal in stroke pathogenesis, particularly if well controlled. Finally, relying on patient health records or patient recollection of adherence to antihypertensive therapy is liable to information and recall bias and thus an unreliable method of determining blood pressure control. Our primary aim was to investigate the magnitude, correlates and outcomes of LVH as a surrogate maker for chronic uncontrolled hypertension in young adults ≤ 45 years with stroke. Our secondary aim was to determine the accuracy of electrocardiography using Sokolow-Lyon and Cornell criteria in detecting LVH compared to echocardiography.

## Methods

### Study design and population

This prospective cohort study was conducted at Muhimbili University of Health and Allied Sciences Academic Medical Center (MAMC), medical wards in Dar es Salaam, Tanzania. MAMC is a tertiary teaching hospital that offers super specialized medical care to all specialties and receives referral patients from both public and private hospitals from all over the country.

We recruited consecutive young stroke participants admitted to MAMC with a clinical diagnosis of first ever stroke as classified by the World Health Organization (WHO) [14] between June 2018 to January 2019. As in previous studies, young adults were defined as those aged between 18 to 45 years [5, 15, 16]. Written informed consent was obtained from participants, or their next of kin if the participant was unable to consent prior to study enrollment.

### Data collection

The principle investigator (SSM) used an interviewer based structured questionnaire and administered it to all study participants or their caregivers capturing the following: Demographics, past medical and drug history, mobile numbers, clinical characteristics, laboratory and imaging studies. All study tools were pre-tested prior to commencing data collection.

### Measurements

#### Demographic characteristics and past medical history

This included the following: Age, gender, residency, marital status and possession of health insurance. Medical information obtained included history of hypertension, diabetes mellitus (DM), cardiac disease, HIV infection and medication use. Patients were dichotomized based on prior use of alcohol or tobacco.

#### Clinical characteristics

This included measurement of blood pressure (BP) using a standard digital BP machine, AD Medical Inc. Three BP readings were collected spaced 5 min apart, while the participant was at rest and an average BP was computed. Hypertension was defined as a systolic blood pressure (SBP) ≥ 140 mmHg or diastolic blood pressure (DBP) ≥ 90 mmHg or a prior diagnosis of hypertension currently on anti-hypertensive therapy [17]. All participants had their waist and hip circumference measured using a tape measure and recorded in centimeters. The waist-hip ratio was interpreted according to the WHO guidelines; in males the ratio of ≥ 0.90 and females ≥ 0.85 was regarded as substantially increased [18]. All the instruments used for measurements were calibrated and results were re-checked before entering data for each study participant.

#### Laboratory investigations

This included collection of capillary fingertip blood samples to check for random blood glucose (RBG) levels and HIV rapid testing using a glucometer GLUCOPLUS™ and SD Bioline respectively. A fasting blood glucose (FBG) sample was collected the following morning for participants with RBG levels of ≥ 11.1 mmol/l. DM diagnosis was defined as a RBG reading of ≥ 11.1 mmol/l, a FBG reading of ≥ 7 mmol/l or a prior diagnosis of DM currently on treatment. For participants who were HIV reactive to SD Bioline, samples were also tested using Unigold Biotech.

We aseptically collected 15mls of venous blood from each study participant and 5mls were analyzed for random total cholesterol and low-density lipoprotein (LDL) using BIO-SYSTEMS machine. Hypercholesteremia was defined as cholesterol > 240 mg/dl and increased LDL was defined as LDL > 129 mg/dl. 5 mls were analyzed for complete blood count using HEMOLYZER 3 PRO machine and 5mls were analyzed for sickling test. Sickling test was performed using sodium metabisulphite and slides were viewed using Olympus microscope. Thrombocytosis was defined as platelets > 450,000 and sickling test results were recorded as either positive or negative. For all tests performed quality control samples were used and tests were verified by a senior laboratory scientist using standard operating procedures for testing and analyzing samples.

#### Brain imaging

Was completed using a non-contrast brain computed tomography scan, GE Healthcare Optima on all study participants on admission and images were interpreted by a senior radiologist as either ischemic or hemorrhagic stroke.

#### Cardiovascular assessment

This included the use of TTE GE Medical Systems and results were interpreted by a qualified cardiologist. All patients were examined in a partial left lateral decubitus position and examinations were performed according to European Society of Cardiology/American Society of Echocardiography [19]. We recorded the left ventricular diameter, valvular structure and functions. Evidence of LVH was defined according to the European Society of Cardiology/American Society of Echocardiography as a measure of severity of septal thickness in 4 chamber view at mid-septum in the end of diastole [20]. A mid septal diameter of 11–13 mm in males and 10–12 mm in females was defined as mild LVH, 14–16 mm in males and 13–15 mm in females as moderate LVH and ≥ 17 mm in males and ≥ 16 mm in females as severe LVH [21]. An ECG using Bionet model Cardio7 machine was performed on the study participants to look for evidence of LVH using the Sokolow-Lyon and Cornell criteria defined as S in V1 plus R in V5 or V6 required to surpass 3.5 mV [22], and the S in V3 plus R in aVL required to surpass 2.0 mV in females and 2.8 mV in males [23] respectively. Atrial fibrillation was defined as the presence of irregular RR intervals and no discernible distinct P waves [24].

#### Stroke outcomes

The National institute of Health Stroke Scale (NIHSS) [14] was used to assess stroke severity on admission. The modified Rankin Scale (mRS) [14] was used to assess post-stroke disability at 24 h, 72 h, 7 days, 14 days and at 30 days from the date of admission, with scores ranging from 0 (no symptoms) to 6 (death). Functional independency was defined as mRS score of 0–2.

### Study variables

The dependent variable was LVH (yes/no) and classified as mild, moderate or severe according to the degree of hypertrophy [21]. The independent variables included: demographic characteristics (age, gender, marital status), risk factors (hypertension, DM, smoking, cardiac disease, alcohol consumption, increased waist-hip ratio, hypercholesteremia, increased LDL, family history of hypertension and diabetes) and outcomes (death or survival with/without disabilities).

### Data analysis

Data was analyzed using SPSS version 20.0. Continuous variables were summarized and presented as means and standard deviation (SD). Categorical variables were summarized as frequencies and proportions. Comparison between proportions were done using Pearson’s Chi square test or Fisher’s exact test. Since the outcome variable (LVH) was common with a proportion of > 10%, logistic regression would have overestimated the odds ratio [25, 26]. We therefore used the modified Poisson regression to determine factors that were independently associated with LVH. All covariates with a *p*-value of < 0.2 in bivariable analysis were included in the multivariable analysis model. Unadjusted and adjusted risk ratios (RR), 95% confidence intervals (CI) and corresponding *p* values were obtained from the models. A two-tailed significance level was set as a *p* value of < 0.05. Receiver operator characteristics (ROC) analysis was performed to estimate the performance of the electrical voltage criterions (Sokolow-Lyon and Cornell criterion) to the gold standard TTE in detecting LVH.

## Results

**Fig. 1:**
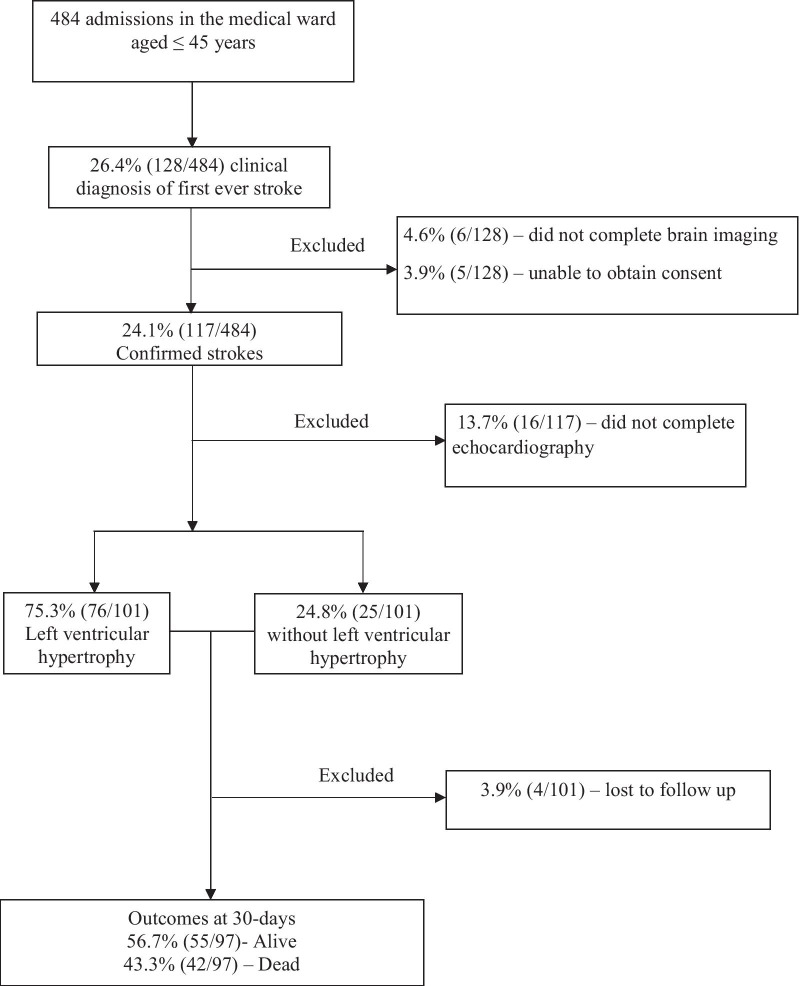
Consort diagram showing the flow of participants

There were a total of 484 medical admissions of patients aged ≤ 45 years between June 2018 to January 2019, of whom 128 (26.4%) participants met the WHO clinical diagnosis of first ever stroke [14]. We excluded 27 (21.1%) participants for the following reasons: unable to consent 5 (3.9%), did not complete brain imaging 6 (4.7%) and did not undergo TTE 16 (12.5%). The remaining 101 (20.9%) young adults with stroke were recruited. There were 4 participants who were lost to follow-up and were excluded from the outcome analysis, Fig. 1.

The mean age ± SD of the recruited participants was 39.7 ± 2 years, and the mean NIHSS score was 18.3 ± 9.2, reflecting severe impairment. The majority of the young participants resided in Dar-es-Salaam 77 (76.2%) and less than a third of the study participants had health insurance 29 (28.7%). The overall mean systolic and diastolic blood pressures at enrollment were 153.5 ± 20.1 mmHg and 94.5 ± 11.4 mmHg (Table 1).

**Table 1:** Baseline characteristics of the young stroke participants, N = 101

Variable	N	%
Female	59	58.4
Mean age ± SD	39.7 ± 2	
Residency		
Dar es Salaam	77	76.2
Marital status		
Ever married	74	73.3
Never married	27	26.7
Insured	29	28.7
Ever smoked	6	5.9
Ever consumed alcohol	23	22.7
Clinical characteristics		
NIHSS Mean ± SD	18.3 ± 9.2	
Systolic blood pressure Mean ± SD	153.5 ± 20.1	
Diastolic blood pressure Mean ± SD	94.5 ± 11.4	

*NIHSS* National institute of health stroke scale, *SD* Standard deviation

Table 2 describes the risk factors for stroke. The majority of the young participants had hypertension (N = 85, 84.2%); 29 (28.7%) were newly diagnosed at hospital admission and 56 (55.4%) were known hypertensives, of who only 17 (30.4%) were on treatment. Other described risk factors include: increased waist-hip ratio seen in 76 (75.2%), use of hormonal contraception in females 29 (49.2%), hypercholesteremia 32 (31.7%), increased LDL 28 (27.7%), DM 15 (14.9%), sickle cell disease 11 (10.9%) and HIV infection 9 (8.9%).

**Table 2:** Description of risk factors for stroke in young adults, N = 101

Variable	N	%
All hypertensive	85	84.2
New	29	28.7
Known	56	55.4
On treatment	17	30.4
Family history of hypertension	40	39.6
All diabetic	15	14.9
New	2	1.9
Known	13	12.9
On treatment	9	69.2
Family history of diabetes	10	9.9
All HIV infected	9	8.9
New	2	1.9
Known	7	6.9
On treatment	7	100
Family history of sudden death	8	7.9
Illicit drug use	4	3.9
Hormonal contraception*	29	49.2
Sickle cell disease	11	10.9
Increased waist-hip ratio	76	75.2
Hypercholesteremia	32	31.7
Increased low density lipoproteins	28	27.7
Thrombocytosis	11	10.9
Valvular heart disease	3	2.9
Atrial fibrillation	3	2.9

*Total females, N = 59

Table 3 summarizes the stroke subtype among the young stroke participants. Ischemic stroke was the major stroke subtype accounting for 60 (59.4%) of all young strokes, 37 (61.7%) were cortical and 23 (38.3%) lacunar infarcts. For those with intracranial hemorrhage, 38 (92.7%) were intracerebral and 3 (7.3%) were subarachnoid. Among those with intracerebral hemorrhage, 33 (86.8%) were located in the basal ganglia reflecting the hypertensive etiology.

**Table 3:** Stroke subtype among young participants, N = 101

Variable	N	%
Ischemic stroke	60	59.4
Cortical	37	61.7
Lacunar	23	38.3
Hemorrhagic stroke	41	40.5
Intra-cerebral	38	92.7
Basal ganglia	33	86.8
Subarachnoid	3	7.3

**Fig. 2:**
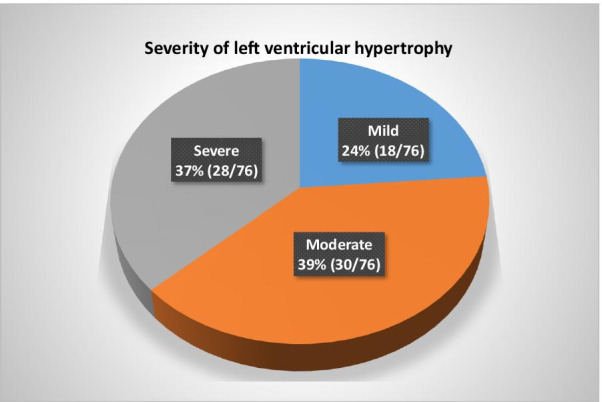
Severity of LVH among young stroke participants

The proportion of LVH confirmed by TTE was 76 (75.3%; 95% CI 65.7%–83.3%). The majority of young stroke participants had moderate 30 (39.5%) or severe 28 (36.8%) degree of LVH, as shown in Fig. 2. Young adults with premorbid and a new diagnosis of hypertension were statistically more likely to have LVH by TTE compared to those without LVH, 47 (61.8%) vs 9 (36%), *p* = 0.024 and 26 (34.2%) vs 3 (12%), *p* = 0.033 respectively, Table 4.

**Table 4:** The proportion of LVH among young hypertensive adults with stroke

Variable	LVH N = 76 (%)	No LVH N = 25 (%)	Total N = 101 (%)	*p* value
Known hypertension	47 (61.8)	9 (36)	56 (55.4)	0.024
New hypertension	26 (34.2)	3 (12)	29 (28.7)	0.033

Correlates for LVH are summarized in Table 5. In bivariable analysis, factors that were significantly associated with LVH among the young stroke participants were: age, hypertension, not on anti-hypertensive medication, family history of hypertension, increased waist-hip ratio, increased LDL and hypercholesteremia. In multivariable analysis after adjusting for other factors, LVH was independently associated with not being on anti-hypertensive medications among hypertensive participants {adjusted RR 1.42 (95% CI: 1.04–1.94)}.

**Table 5:** Factors associated with LVH among young stroke participants

Factor	Total	No. with LVH (%)	Unadjusted RR (95% CI)	*p* value	Adjusted RR (95% CI)	*p* value
Age group (years)						
18–30	11	45.5	1		1	
31–45	90	78.9	1.73 (0.91–3.34)	0.10	0.93 (0.52–1.64)	0.80
Gender						
Female	59	72.2	1			
Male	42	81.0	1.12 (0.91–1.42)	0.25		
Hypertension						
No	45	38.2	1		1	
Yes	56	61.8	1.62 (1.01–1.83)	0.04	0.79 (0.48–1.32)	0.37
Not on anti hypertensives						
No	17	54.9	1		1	
Yes	39	83.0	1.51 (1.02–2.32)	0.01	1.42 (1.04–1.94)	0.03
Family history of hypertension						
No	59	64.4	1		1	
Yes	42	90.5	1.41 (1.14–1.74)	0.002	1.13 (0.92–1.39)	0.26
Diabetes						
No	88	75.0	1			
Yes	13	76.9	1.03 (0.74–1.41)	0.88		
Smoking						
No	95	74.7	1			
Yes	6	83.3	1.12 (0.77–1.63)	0.57		
Atrial fibrillation						
No	88	75.0	1			
Yes	3	66.7	0.89 (0.39–1.99)	0.78		
Increased waist-hip ratio						
No	25	46.2	1		1	
Yes	76	79.5	1.72 (0.95–3.13)	0.07	1.22 (0.74–2.02)	0.43
Increased LDL						
No	73	73.8	1		1	
Yes	28	84.0	1.14 (0.95–1.46)	0.13	1.03 (0.83–1.27)	0.81
Hypercholesteremia						
No	69	72.7	1		1	
Yes	32	89.3	1.23 (1.05–1.55)	0.01	1.08 (0.87–1.33)	0.49

**Fig. 3:**
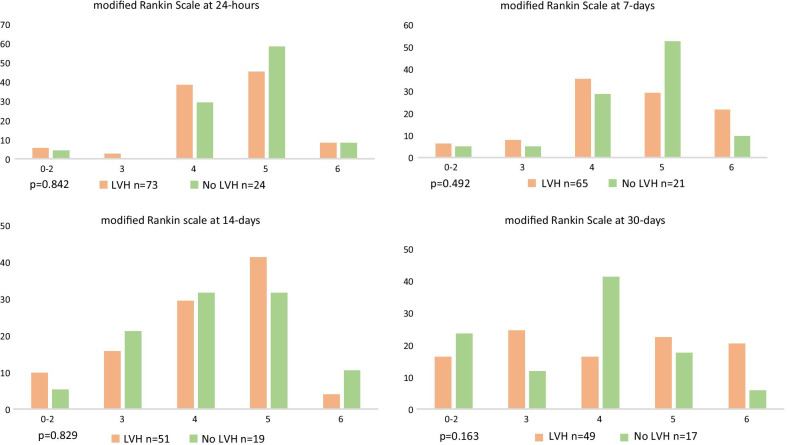
Bar chart demonstrating proportional spread of mRS at multiple time points

Figure 3 describes the mRS scores at 24 h, 7-days, 14-days and 30-days after excluding lost to follow-up. The overall mortality at 30 days was 42 (43.3%), with no statistical significant difference in mortality between young strokes with and without LVH. A total of 55 (56.7%) participants survived to 30-days, of whom 8 (16.3%) and 4 (23.5%) of patients with LVH and without LVH respectively regained functional independence.

**Fig. 4:**
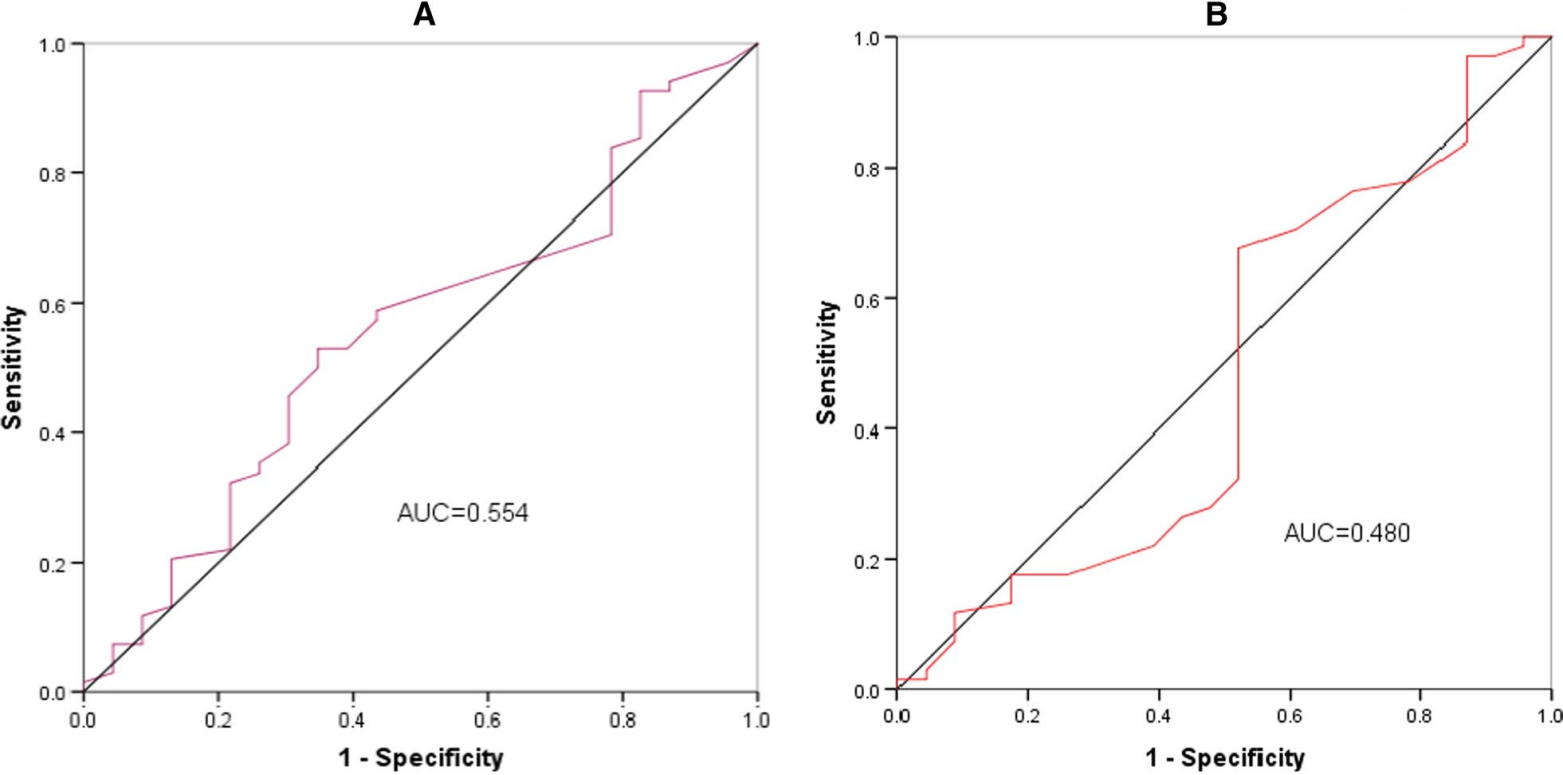
ROC and AUC of **A** Sokolow-Lyon criteria and **B** Cornell criteria for detecting LVH

The sensitivity and specificity analysis for the detection of LVH using the electrical voltage criteria (Sokolow-Lyon and Cornell criteria) are presented in Table 6. ECG could not be performed on 10 young stroke participants and were excluded from this analysis. The sensitivity and specificity for the Sokolow-Lyon criteria in detecting LVH was 27% and 78% and for Cornell criteria was 32% and 52% respectively. The ROC analysis was also carried out and the results are shown in Fig. 4. Both criterions did not reach statistical significance, for Sokolow-Lyon criteria area under the curve (AUC) = (0.55 [95% CI 0.42–0.69], *p* = 0.44) and Cornell criteria AUC = (0.48 [95% CI 0.33–0.63], *p* = 0.78) (Fig. 4).

**Table 6:** The sensitivity and specificity of the electrical voltage criteria in detecting LVH

	Sokolow-Lyon criteria	Cornell criteria
True positive (n)	18	22
True negative (n)	18	12
False positive (n)	5	11
False negative (n)	50	46
Sensitivity (%) 95% CI	0.27 (0.17–0.39)	0.32 (0.22–0.45)
Specificity (%) 95% CI	0.78 (0.56–0.92)	0.52 (0.31–0.73)

## Discussion

Our primary aim was to investigate the magnitude, correlates and outcomes of left ventricular hypertrophy as a surrogate maker for chronic uncontrolled hypertension in young Tanzanian adults (≤ 45 years). We also aimed to determine the accuracy of electrocardiography using Sokolow-Lyon and Cornell criteria in detecting left ventricular hypertrophy compared to echocardiography.

We found that three-quarters of young participants with stroke had LVH as diagnosed by TTE. This proportion of LVH is higher than that described in other studies: In the United States, Bruno et al., found up to 46% of stroke patients with known hypertension were found to have LVH by TTE [27], Luciana et al. found 30% of young adults with first ever stroke had LVH on TTE [28] and Levy et al. described a prevalence of 6% among adults under 30 years [29]. It is notable that our study was different, since it exclusively included an African population. Numerous reports have demonstrated that ethnicity plays an important role in the epidemiology of LVH [12, 30, 31]. Compared to whites, Africans with or without stroke are more likely to have LVH across all age groups. Adeoye et al. in West Africa found that in a cohort of stroke participants above 18 years, younger age (≤ 45 years), female gender and uncontrolled hypertension was independently associated with LVH [11].

The high proportion of patients with LVH in our cohort suggests that chronic uncontrolled hypertension is a significant contributor to stroke occurrence in young adults. Furthermore, hypertension was observed in 84.2% of the study participants and was the key risk factor. Young adults with a ‘new’ diagnosis of hypertension at hospital admission were statistically more likely to have LVH on TTE (seen in 34.2%). Likewise, young stroke participants with premorbid hypertension were statistically more likely to have LVH (present in 61.8%), again suggesting poor blood pressure control since; firstly, the majority had moderate and severe degrees of hypertrophy. Secondly, we found that LVH was independently associated with not being on anti-hypertensive medications among hypertensive participants. The causes of non-adherence to treatment are multi-factorial and are likely to include a lack of insurance, which was the case in two-thirds of the study participants.

Longitudinal studies have established that LVH progresses from young adulthood to middle age and is compounded by hypertension, DM and tobacco smoking [32]. Similarly, several studies have demonstrated an increased risk of stroke among individuals with LVH secondary to hypertension [33, 34].

In this study more than two-thirds (86.8%) of the young stroke participants had deep basal ganglia intracerebral hemorrhage on brain CT, in keeping with the known pathophysiology of this stroke subtype. Hypertensive hemorrhages typically occur in these regions as penetrating arteries are susceptible to hypertensive rupture [35]. These findings further support that hypertension in young adults is not an uncommon risk factor for stroke.

Hypertension is modifiable and currently on the rise in the young population in SSA, and is mainly attributed to rapid transitioning and urbanization [36]. Our cohort is a classic example of a population undergoing rapid epidemiological transitioning, with more than two-thirds of the study participants residing in Dar-es-Salaam, a former capital city and therefore are more likely to adopt the western way of living. In this study, we found that the traditional risk factors (hypertension and obesity) were the main risk factors for stroke in this young population, similar to those observed in the elderly stroke population. This is contrary to more common stroke etiologies in young adults (e.g. dissections, paradoxical embolism, infective causes of stroke, thrombophilias, etc.) [37–39]. This merits the need for early screening to detect hypertension during the adolescent period at secondary schools, long before multiple end organ damage manifests. Further research is needed to investigate the etiologies of hypertension in young African adults.

In this study almost half of the participants died within 30 days with no statistical significant difference between those with and without LVH. Therefore, we believe primary prevention is the main route to reducing the stroke burden from chronic uncontrolled hypertension. Once young adults succumb to stroke, their prognosis is generally poor and leads to a decline in the family and nation’s economy.

Given the cost and availability of TTE, we also aimed to assess the performance of the electrical voltage criteria for detecting LVH compared to TTE. We found that both the Sokolow-Lyon and Cornell criteria had a low sensitivity, moderate specificity and poor performance in detecting LVH in our population, making it a less accurate and unreliable method. Therefore, the key public health message from this research is that efforts should be centered at promoting low cost interventions such as blood pressure screening, treatment and control which are likely to reduce the burden of stroke in young Africans.

Our study had the following limitations: It was a single center with a small sample size and underpowered to determine the diagnostic accuracy of ECG for LVH, and therefore limits generalizability. There were a few participants (3.9%) with LVH with no evidence of hypertension therefore other etiologies (e.g. genetics) could be responsible. However, the majority of participants had premorbid hypertension (making hypertrophic obstructive cardiomyopathy as a cause of LVH unlikely) and no participant had stigmata of other differentials, such as Fabry’s disease.

## Conclusions

We identified a high proportion of left ventricular hypertrophy in young adults with stroke associated with chronic undertreated hypertension. While the study methodology does not allow us to determine causation, this association and current knowledge of pathophysiological processes supports the notion that chronic hypertension is a major risk factor for young strokes which is associated with high mortality. Our findings did not support the use of the electrical voltage criteria for detecting left ventricular hypertrophy. We recommend low cost interventions such as blood pressure screening, treatment and control to reduce this burden.

## Data Availability

Data are available from the corresponding author on reasonable request.

## Abbreviations

AF: Atrial fibrillation
DALYS: Disability adjusted life years
DWI: Diffusion weighted image
FBG: Fasting blood glucose
HIV: Human deficiency virus
mRS: Modified Rankin Scale
NIHSS: National Institute of Health Stroke Scale
SSA: Sub Saharan Africa
TTE: Trans thoracic echocardiography
WHO: World Health Organization

## References

[1] Krishnamurthi RV, Ikeda T, Feigin VL (2020). Global, regional and country-specific burden of ischaemic stroke, intracerebral haemorrhage and subarachnoid haemorrhage: a systematic analysis of the global burden of disease study 2017. Neuroepidemiology.

[2] O’Donnell MJ, Chin SL, Rangarajan S, Xavier D, Liu L, Zhang H (2016). Global and regional effects of potentially modifiable risk factors associated with acute stroke in 32 countries (INTERSTROKE): a case-control study. Lancet.

[3] Willey JZ, Moon YP, Kahn E, Rodriguez CJ, Rundek T, Cheung K, et al. Population attributable risks of hypertension and diabetes for cardiovascular disease and stroke in the Northern Manhattan study. J Am Heart Assoc. 2014;3(5). 10.1161/JAHA.114.001106 PMC4323833 25227406

[4] Walker RW, Jusabani A, Aris E, Gray WK, Unwin N, Swai M (2013). Stroke risk factors in an incident population in urban and rural Tanzania: a prospective, community-based, case-control study. Lancet Glob Heal.

[5] Matuja SS, Munseri P, Khanbhai K (2020). The burden and outcomes of stroke in young adults at a tertiary hospital in Tanzania: a comparison with older adults. BMC Neurol.

[6] Obiako O, Ogunniyi A, Oparah S (2011). Prognosis and outcome of acute stroke in the University College Hospital Ibadan, Nigeria. Niger J Clin Pract.

[7] Deresse B, Shaweno D. Epidemiology and in-hospital outcome of stroke in South Ethiopia. J Neurol Sci. 2015;355(1–2). 10.1016/j.jns.2015.06.001 26059446

[8] Desai CS, Bartz TM, Gottdiener JS, Lloyd-Jones DM, Gardin JM (2016). Usefulness of left ventricular mass and geometry for determining 10-year prediction of cardiovascular disease in adults aged >65 years (from the cardiovascular health study). Am J Cardiol.

[9] Marwick TH, Gillebert TC, Aurigemma G, Chirinos J, Derumeaux G, Galderisi M (2015). Recommendations on the use of echocardiography in adult hypertension: a report from the European Association of Cardiovascular Imaging (EACVI) and the American Society of Echocardiography (ASE). J Am Soc Echocardiogr.

[10] Chillo P, Lwakatare J, Rieck ÅE, Lutale J, Gerdts E (2014). Prevalence and covariates of abnormal left ventricular geometry in never-treated hypertensive patients in Tanzania. Blood Press.

[11] Adeoye AM, Ovbiagele B, Kolo P, Appiah L, Aje A, Adebayo O (2017). Exploring overlaps between the genomic and environmental determinants of LVH and stroke: a multicenter study in West Africa. Glob Heart.

[12] Drazner MH, Dries DL, Peshock RM, Cooper RS, Klassen C, Kazi F (2005). Left ventricular hypertrophy is more prevalent in blacks than whites in the general population: the Dallas heart study. Hypertension.

[13] Quispe-Tintaya W (2017). HHS public access. Physiol Behav.

[14] WHO Noncommunicable Diseases and Mental Health. The WHO STEPwise approach to stroke surveillance report. 2005.

[15] Park W-B, Cho J-S, Kong S-Y, Kim J-J, Lim Y-S, Yang H-J (2014). Comparison of epidemiology, emergency care, and outcomes of acute ischemic stroke between young adults and elderly in Korean Population: a multicenter observational study Sang-Do Shin. J Korean Med Sci.

[16] Miah M, Azhar M, Rahman A, Halder D, Akteruzzaman M, Kundu N (2012). Risk factors of stroke in young and old age group—a comparative study. J Med.

[17] Chobanian AV, Bakris GL, Black HR, Cushman WC, Green LA, Izzo JL, et al. Seventh report of the Joint National Committee on Prevention, Detection, Evaluation, and Treatment of High Blood Pressure. Vol. 42, Hypertension. Lippincott Williams & Wilkins; 2003. p. 1206–52. 10.1161/01.HYP.0000107251.49515.c2 14656957

[18] World Health Organization. Waist Circumference and Waist-Hip Ratio: Report of a WHO Expert Consultation. Who. 2011;(8–11 December 2008):1–39.

[19] Kim M, Kim HL, Park KT, Kim YN, Lim JS, Lim WH (2020). Echocardiographic parameters determining cardiovascular outcomes in patients after acute ischemic stroke. Int J Cardiovasc Imaging.

[20] Lang R, Bierig M, Devereux R, Flachskampf F, Foster E, Pellikka P (2006). Recommendations for chamber quantification☆. Eur J Echocardiogr.

[21] Lang RM, Badano LP, Victor MA, Afilalo J, Armstrong A, Ernande L (2015). Recommendations for cardiac chamber quantification by echocardiography in adults: an update from the American Society of Echocardiography and the European Association of Cardiovascular Imaging. J Am Soc Echocardiogr.

[22] Sokolow M, Lyon TP (1949). The ventricular complex in left ventricular hypertrophy as obtained by unipolar precordial and limb leads. Am Heart J.

[23] Casale P, Devereux R, Kligfield P, Eisenberg R, Miller D, Chaudhary B (1985). Electrocardiographic detection of left ventricular hypertrophy: development and prospective validation of improved criteria. JAA.

[24] Benussi S (2016). 2016 ESC Guidelines for the management of atrial fibrillation developed in collaboration with EACTS. Eur Heart J.

[25] Barros AJD, Hirakata VN (2003). Alternatives for logistic regression in cross-sectional studies: an empirical comparison of models that directly estimate the prevalence ratio. BMC Med Res Methodol.

[26] Zou G (2004). A modified Poisson regression approach to prospective studies with binary data. Am J Epidemiol.

[27] Bruno A, Brooks DD, Abrams TA, Poorak MD, Gunio D, Kandhal PK (2017). Left ventricular hypertrophy in acute stroke patients with known hypertension. Clin Exp Hypertens.

[28] Catanese L, Shoamanesh A, Lau H, Romero J, Babikian V, Kase C, et al. Left ventricular hypertrophy as a predictor of white matter hyperintensities in young stroke patients (P2.111). Neurology. 2014;82(10 Supplement):111.

[29] Levy D, Anderson KM, Savage DD, Kannel WB, Christiansen JC, Castelli WP (1988). Echocardiographically detected left ventricular hypertrophy: Prevalence and risk factors. The Framingham heart study. Ann Intern Med.

[30] Yan Hou, Elizabeth Aradine, Kathleen Ryan, Prachi Mehndiratta, Seemant Chaturvedi, Carolyn A Cronin, Michael S Phipps, Marcella A Wozniak, Jose G Merino-Juarez, Tara M Dutta, John W Cole SJK. African American Young Adults with Ischemic Stroke Have High Rates of Left Ventricular Hypertrophy | Stroke [Internet]. American Stroke Association. 2020 [cited 2020 Dec 11]. p. 200. Available from: 10.1161/str.51.suppl_1.TP200.

[31] Kamath S, Markham D, Drazner MH (2006). Increased prevalence of concentric left ventricular hypertrophy in African-Americans: Will an epidemic of heart failure follow?. Heart Fail Rev.

[32] Gidding SS, Liu K, Colangelo LA, Cook NL, Goff DC, Glasser SP (2013). Longitudinal determinants of left ventricular mass and geometry: The coronary artery risk development in young adults (CARDIA) study. Circ Cardiovasc Imaging.

[33] Verdecchia P, Angeli F, Gattobigio R, Sardone M, Pede S, Reboldi GP (2006). Regression of left ventricular hypertrophy and prevention of stroke in hypertensive subjects. Am J Hypertens.

[34] Bots ML, Nikitin Y, Salonen JT, Elwood PC, Malyutina S, Freire de Concalves A, et al. Left ventricular hypertrophy and risk of fatal and non-fatal stroke. EUROSTROKE: a collaborative study among research centres in Europe. J Epidemiol Community Health. 2002;56(1):18–13. 10.1136/jech.56.suppl_1.i8 PMC1765512 11815638

[35] Jh G, Ho K (1992). Pathology of hypertensive arteriopathy—PubMed. Neurosurg Clin N Am.

[36] Guwatudde D, Nankya-Mutyoba J, Kalyesubula R, Laurence C, Adebamowo C, Ajayi I (2015). The burden of hypertension in sub-Saharan Africa: a four-country cross sectional study. BMC Public Health.

[37] Ovbiagele B, Nath A (2011). Increasing incidence of ischemic stroke in patients with HIV infection. Neurology.

[38] Marini C, Russo T, Felzani G (2010). Incidence of stroke in young adults: a review. Stroke Res Treat.

[39] Yahya T, Jilani MH, Khan SU, Mszar R, Hassan SZ, Blaha MJ (2020). Stroke in young adults: Current trends, opportunities for prevention and pathways forward. Am J Prev Cardiol.

